# Proximate Causes of Hemorrhage

**Published:** 1844-04

**Authors:** 


*Proximate Cause of Hæmorrhage.*—M. Andral, in his recently published Essay on Hæmatology, states it, as the result of his experimental researches, that some hæmorrhages are always connected with, if not immediately dependent on, a state of blood in which there is an excess of red globules; while others are very usually associated with an *hæmatosis*, characterized by a deficiency of the fibrine in the circulating fluid. The former are observed to occur in persons of a plethoric constitution; but they are far from being either as frequent, or as serious, as the second sort of the disease, such as takes place in scurvy and similar diseases. Is not this division of hæmorrhages very much the same as that adopted by the old writers, viz: into *active* and *passive?*—a division that is incompatible indeed with certain theories of modern times, but one with which neither clinical medicine nor therapeutic instruction can dispense.

(The remark made here is quite just. There are several kinds of hæmorrhage, just as there are several kinds of fever; it cannot therefore be wonderful that the same treatment is not appropriate in all.. One case may require bleeding, and nauseating purgatives; while another demands the use of powerful tonics and astringents. The *internal* exhibition of alum is, we think, too much overlooked in the present day, as a powerful means of arresting many hæmorrhages).—*Med. Chi. Rev.*, Jan.

